# Nunavut: A Health System Profile

**DOI:** 10.3402/ijch.v72i0.22877

**Published:** 2013-10-10

**Authors:** Gregory P. Marchildon, Renée Torgerson

Gregory P. Marchildon and Renée Torgerson. *Nunavut: A Health System Profile*. Montreal: McGill-Queen's University Press, 2013. [ISBN: 9780773541481]


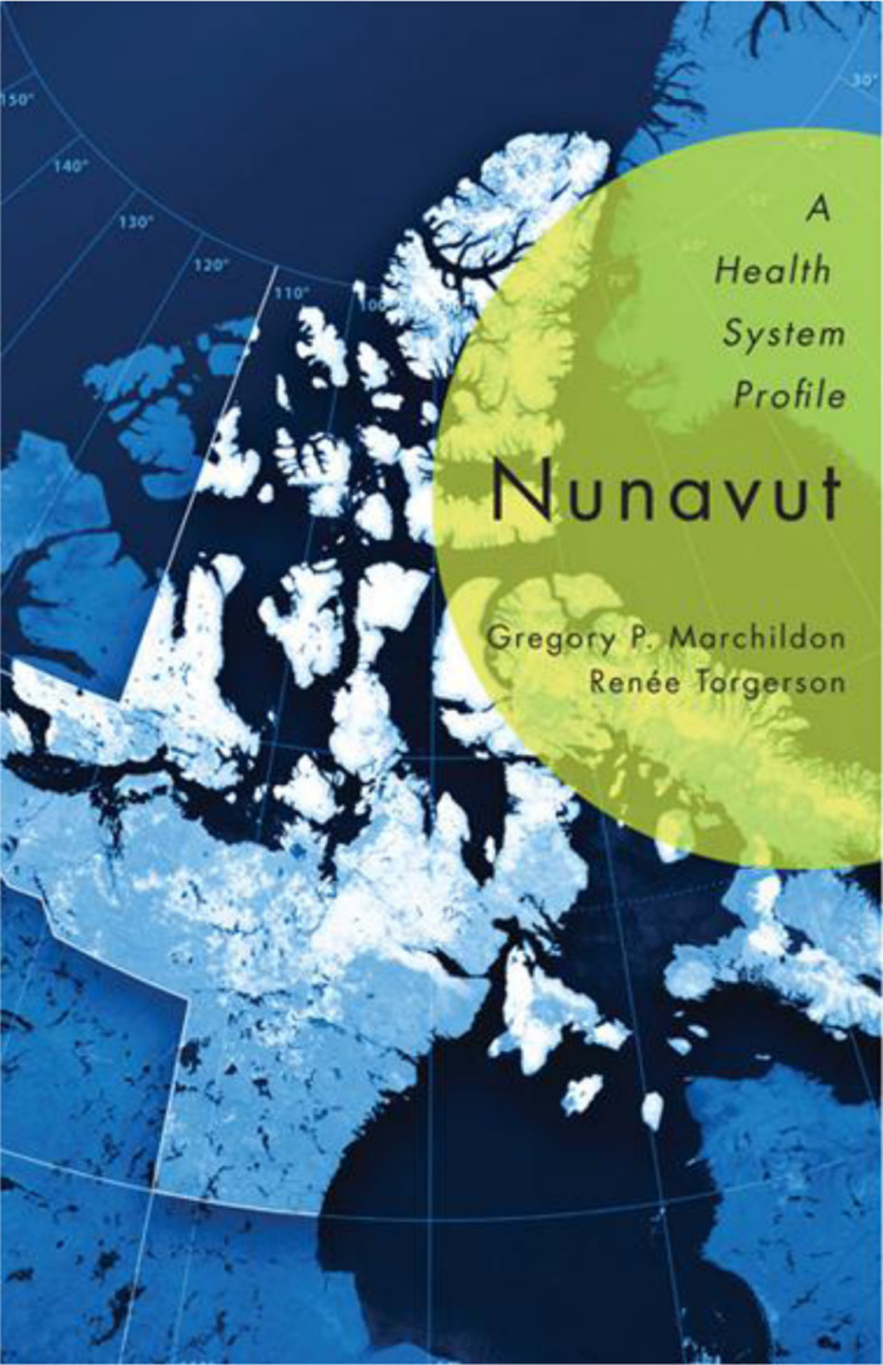


Based on extensive research, including visits to most health centres and facilities in Nunavut, Gregory P. Marchildon and Renée Torgerson have produced a comprehensive review of healthcare in Canada's newest territory. *Nunavut: A Health System Profile* provides an in-depth examination of population health and healthcare in the territory. Little more than a decade old, Nunavut has a population that consists of 30,000 residents living in 25 widely dispersed communities. No roads connect the territory's isolated populations and nearly all supplies and equipment are transported by air. Consequently, health service delivery in Nunavut is the costliest in Canada and its operation encounters challenges more extreme than those faced elsewhere. Marchildon and Torgerson consider the historical and demographic context of healthcare in Nunavut, as well as the finances, governance, infrastructure, workforce and programme provisions that define the system. Due to a high incidence of suicide and the psychological upheaval associated with rapid societal change, the authors call particular attention to the treatment of mental health and addictions. Filling a gap in our understanding of one of Canada's most important and expensive social policies, *Nunavut: A Health System Profile* provides the first comprehensive review of the health system in Nunavut and the distinct health issues the territory faces.

Order at: http://www.mqup.ca/nunavut-products-9780773541474.php?page_id=73&#!prettyPhoto

